# Recent development of contrast agents for magnetic resonance and multimodal imaging of glioblastoma

**DOI:** 10.1186/s12951-022-01479-6

**Published:** 2022-06-16

**Authors:** Danping Zhuang, Huifen Zhang, Genwen Hu, Bing Guo

**Affiliations:** 1 The Second Clinical Medical College, Jinan University, Shenzhen, Guangdong 518020 China; 2 Department of Radiology, Shenzhen People’s Hospital (The Second Clinical Medical College, Jinan University; The First Affiliated Hospital, Southern University of Science and Technology), Shenzhen, 518020 Guangdong China; 3 School of Science and Shenzhen Key Laboratory of Flexible Printed Electronics Technology, Harbin Institute of Technology, Shenzhen, 518055 China

**Keywords:** Glioblastoma, MRI, blood–brain barrier, Contrast agents

## Abstract

Glioblastoma (GBM) as the most common primary malignant brain tumor exhibits a high incidence and degree of malignancy as well as poor prognosis. Due to the existence of formidable blood–brain barrier (BBB) and the aggressive growth and infiltrating nature of GBM, timely diagnosis and treatment of GBM is still very challenging. Among different imaging modalities, magnetic resonance imaging (MRI) with merits including high soft tissue resolution, non-invasiveness and non-limited penetration depth has become the preferred tool for GBM diagnosis. Furthermore, multimodal imaging with combination of MRI and other imaging modalities would not only synergistically integrate the pros, but also overcome the certain limitation in each imaging modality, offering more accurate morphological and pathophysiological information of brain tumors. Since contrast agents contribute to amplify imaging signal output for unambiguous pin-pointing of tumors, tremendous efforts have been devoted to advances of contrast agents for MRI and multimodal imaging. Herein, we put special focus on summary of the most recent advances of not only MRI contrast agents including iron oxide-, manganese (Mn)-, gadolinium (Gd)-, ^19^F- and copper (Cu)-incorporated nanoplatforms for GBM imaging, but also dual-modal or triple-modal nanoprobes. Furthermore, potential obstacles and perspectives for future research and clinical translation of these contrast agents are discussed. We hope this review provides insights for scientists and students with interest in this area.

**Graphical abstract:**

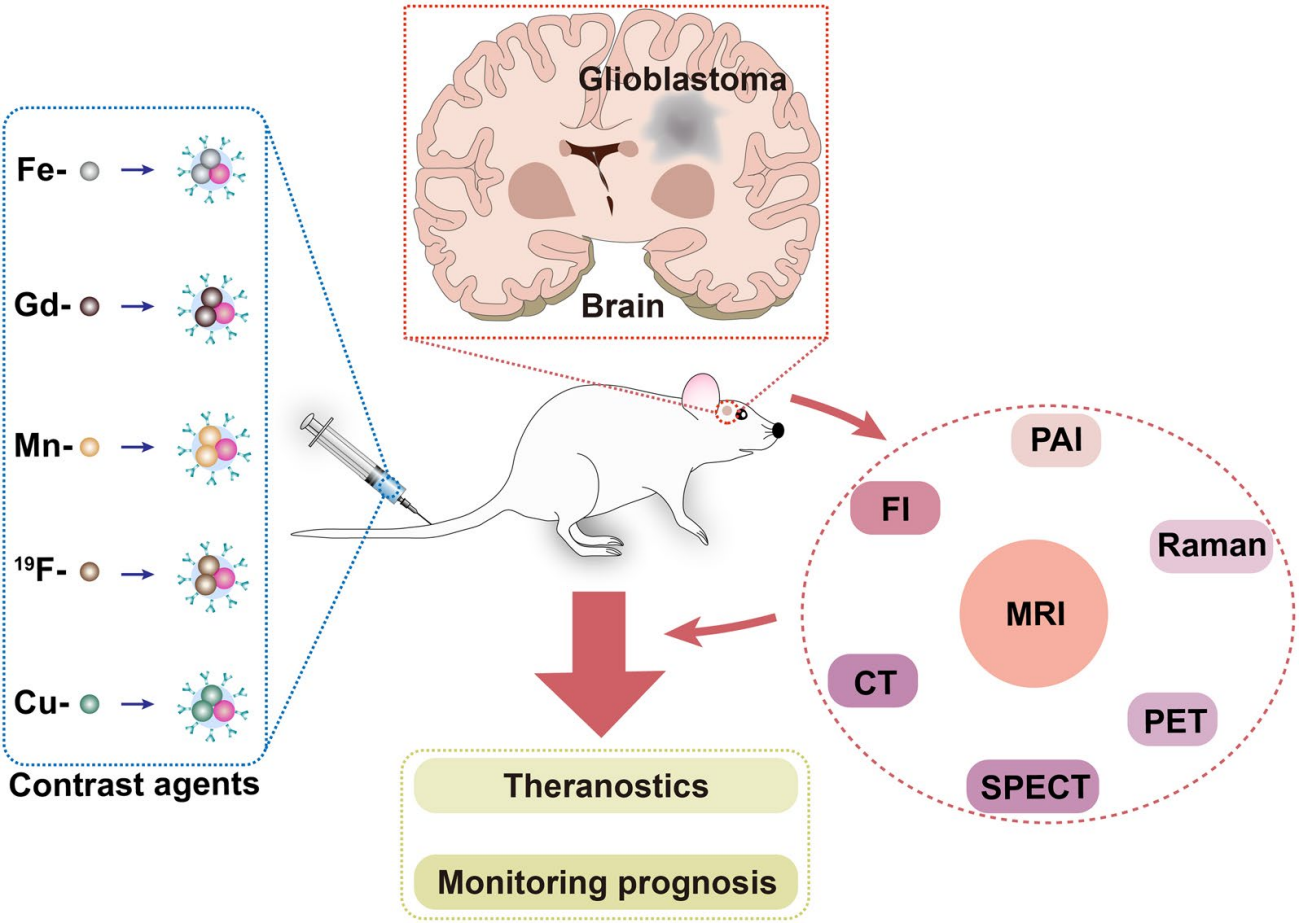

## Introduction

Glioblastoma (GBM), a grade 4 glioma, is the most common primary malignant brain tumor with the medium survival of 8 months regardless of treatment [1, 2]. The standard treatment for glioma is composed of maximum surgical excision, and subsequent image-guided radiotherapy and chemotherapy, but the prognosis remains poor because the highly aggressive nature of the tumor makes complete surgical excision impossible and it is often prone to recurrence at the site of surgery [3].

At present, magnetic resonance imaging (MRI) has become the preferred tool for GBM diagnosis owing to its unique merits of non-invasiveness, non-limited penetration depth, high resolution and soft-tissue contrast. Structural MRI sequences with a magnetic field of 1.5 T or more are generally employed to diagnose brain tumors and monitor the therapeutic tactics in clinic [4]. There are a variety of MRI sequences, among which the commonly used imaging sequences are T1-weighted MRI (T1WI) and T2-weighted MRI (T2WI). T1WI is able to better display the anatomical structure of various brain tissues, while T2WI can identify lesions and determine tumor types. In order to further improve resolution and sensitivity of the scans, so-called contrast agents are often used. For example, the boundary information of brain tumors can be observed more clearly with the assistance of gadolinium (Gd)-based T1 contrast agents [5]. On the other hand, the FDA-approved 30-nm magnetic nanoparticles (MNPs) have been used to predict the co-localization of therapeutic nanoparticles (NPs) with tumors by MRI, and changes in mean T2 mapping are utilized to quantify MNP levels [6]. In addition to MRI, computed tomography (CT), positron emission tomography (PET), single photon emission CT (SPECT), fluorescence imaging (FI), photoacoustic imaging (PAI), and Raman imaging have already been utilized to detect GBM [7, 8]. As with MRI, CT provides anatomical structure information [9, 10]. PET and SPECT are able to measure the metabolic or enzymatic processes through injection of radiolabelled tracers, creating the most accurate quantitative maps for the metabolism in the target region [11]. FI and PAI allow real-time imaging, and notably the second near-infrared (NIR-II) FI is capable of providing deeper penetration depths and improving imaging fidelity in contrast to the first near-infrared (NIR-I) FI [12]. For Raman imaging, it possesses high resolution, excellent photostability and ignorable autofluorescence [13]. However, these imaging modalities alone have certain drawbacks, such as the long acquisition time and low spatial coverage for MRI, hazardous ionizing radiation for CT, PET and SPECT, limited penetration for FI and Raman, and restricted imaging area for PAI [13–16].

In short, single-modal imaging cannot satisfy the increasing demands on the accuracy and efficiency for clinical diagnosis or medical research [17]. Therefore, the combination of MRI with other detection techniques has turned to be the research hotpot in recent years, aiming to complement each other and achieve more accurate morphological and pathophysiological information of GBM [18]. More importantly, these multifunctional contrast agents can also be endowed with the following advantages, including low toxicity, high biocompatibility, especially the abilities of the blood–brain barrier (BBB) crossing and efficient tumor targeting and as well as therapeutic units. In this work, we address the composition of the BBB and the blood-tumor barrier (BTB), discuss the pathways for crossing the BBB and review the recent advances in diverse nanoplatforms for MRI and MRI-based multimodal imaging of GBM.

## BBB and BTB

**Fig. 1:**
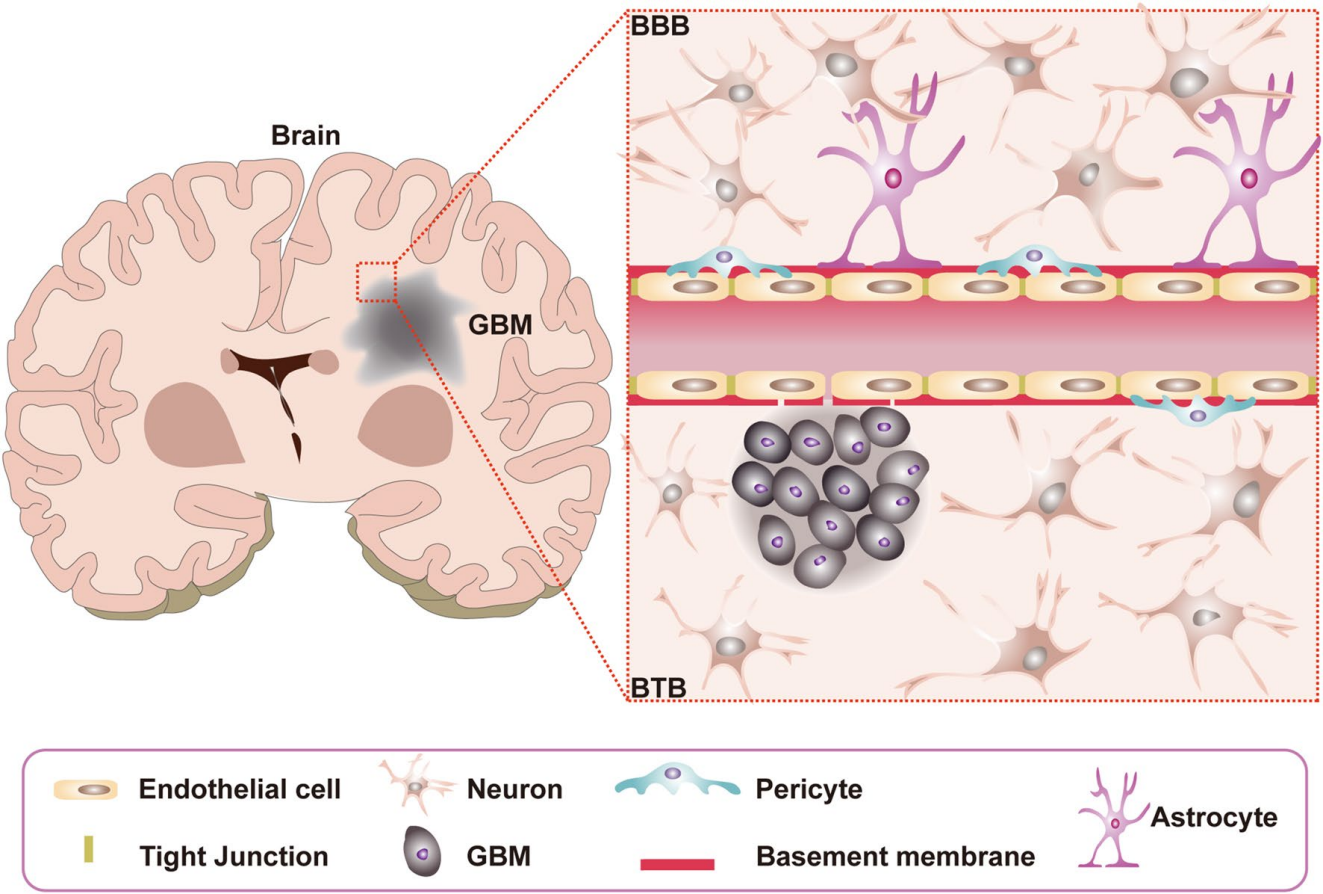
Schematic representation of capillaries in the intact BBB and the BTB in the brain

The BBB consists of five components including pericytes, astrocytes, neurons, basement membrane, and junctional complexes which involve mainly endothelial cells (ECs) and as well as tight junctions (TJs) (Fig. 1) [19]. Among them, pericytes are embedded in the basement membrane of blood vessels, which possess numerous vital functions including adjustment of cerebral blood flow, maintenance of the BBB, and regulation of angiogenesis [20]. For astrocytes, they are located between neurons and ECs, and play an important role in neurotrophic support and regulation of cerebral blood flow. Besides, astrocytes restrict peripheral immune cells from crossing the BBB under physiological conditions [21]. For basement membrane, it forms the extracellular matrix surrounding the vascular vessels and pericytes, and closely contacts with the end-feet of astrocytes. In addition, basement membrane performs many essential functions such as structural support, cell anchoring and signal transduction [22, 23]. For TJs, they are located among ECs, and contribute to force most molecular transport to take a trans-cellular route through the BBB rather than para-cellular route. The structure of the BBB allows the entry of desired nutrients and the excretion of potentially harmful compounds. It is necessary for brain homeostasis and normal neuronal function [24, 25]. Tumors would damage the integrity of the BBB to form BTB, which is characterized by the loss of connections between astrocyte endings and neurons, abnormal distribution of pericytes, and disruption of TJs, but retains ECs and expression of active efflux transporters in tumor cells (Fig. 1) [26, 27]. Although BTB is more permeable than BBB, the molecules are still unevenly distributed in the tumor [28]. In addition, even for brain tumors at their late stage, the Gd permeability is lower than that in normal organs outside of brain [29].

## Pathway for crossing the BBB/BTB

**Fig. 2:**
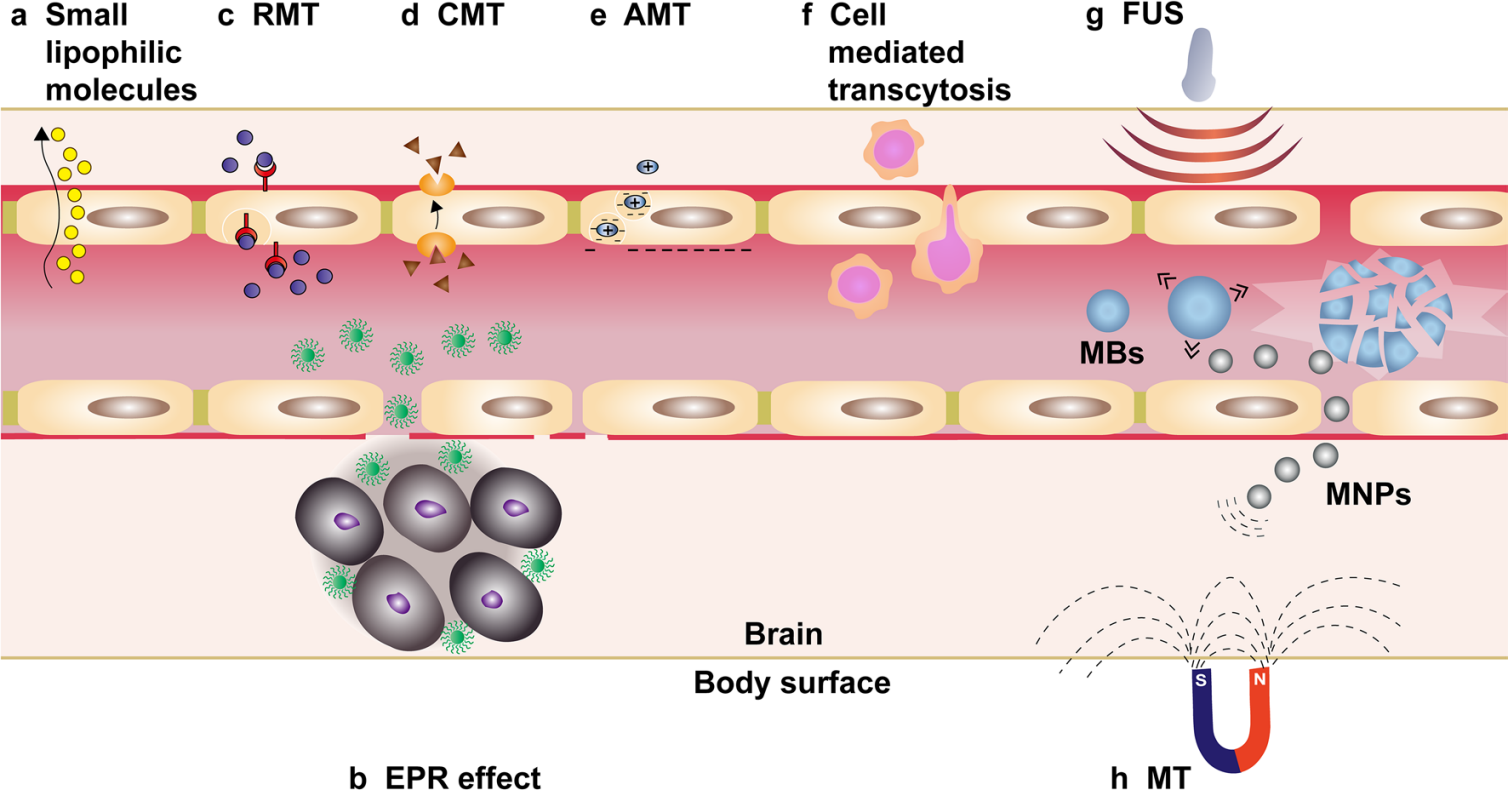
Schematic illustration of routes for molecular traffic across the BBB. **a** Schematic imaging of diffusion of lipophilic small molecules into the brain. **b** Schematic imaging of NPs can traverse BTB via EPR effect. **c**–**e** show respectively NPs penetrating BBB through RMT, CMT and AMT. **f** Schematic imaging of cell-mediated transcytosis. Schematic imaging of MBs open the BBB reversibly under the FUS shows in **g** and the subsequent application of MT significantly improves deposition of therapeutic MNPs shows in **h**

The composition of the BBB excludes 100% of large-molecule drugs and over 98% of all small-molecule drugs from the brain [30]. Only small lipophilic molecules (< 500 Da) can cross the BBB at therapeutic concentrations (Fig. 2a) [31]. The increased permeability of tumor vessels leads to the accumulation of macromolecules and NPs in the tumor due to the enhanced permeability and retention (EPR) effect, a general pathophysiological phenomenon and mechanism that largely depends on the type and location of the tumor (Fig. 2b) [32]. Meanwhile, the EPR effect is affected by the particle size and pathological conditions in the disease tissues. The previous experimental results suggest that only MNPs with size of less than 50 nm could reach the tumor mesenchyme, while larger MNPs were not able to pass through the BTB [33]. Notably, for MNPs even with a diameter of 30–50 nm, only a few portion of the MNPs accumulate inside tumor, while most part of the MNPs stay in vicinity vasculatures surrounding the tumor [33]. Therefore, researchers have been devoted to investigating brain-targeted NPs delivery strategies such as receptor-mediated transcytosis (RMT), carrier-mediated transcytosis (CMT), adsorptive-mediated transcytosis (AMT), cell-mediated transport, and BBB disruption-enhanced transport.

RMT is a vesicular trafficking machinery of ECs to transport endogenous nutrients required for brain function to across the BBB (Fig. 2c) [34]. It crosses the ECs of the BBB in four main steps including targeted binding of ligand to receptor, endocytosis, intracellular transport, and exocytosis [35]. Several major receptors like transferrin receptor (TfR), insulin and insulin-like growth factors receptors, low-density lipoprotein receptor (LDLR), and neuropeptide receptors have been reported for RMT [36]. For instance, Liang et al. [37] constructed MnO_2_@Tf-ppIX (TMP) NPs using holo-transferrin (holo-Tf), MnO_2_ nanocrystals and protoporphyrin (ppIX). Among them, Tf could target TfR to across the BBB and target GBM. MnO_2_ served as MRI contrast agent and catalase-mimicking nanozyme to catalyze H_2_O_2_ to produce O_2_ in the tumor microenvironment (TME). Under ultrasound (US) irradiation, sonosensitizer ppIX generated singlet oxygen (^1^O_2_) for sonodynamic therapy. More importantly, the experiment showed that the TMP NPs have good biosafety and potential for clinical translation. For another example, a dual-targeting probe was used for preoperative and intraoperative imaging as follows. By incorporating indocyanine (Cy7) molecules with retro-enantio isomer of angiopep-2 (^D^ANG) modified superparamagnetic iron oxide NPs (SPIONs), Xie et al. [38] developed a ^D^ANG/Cy7-SPIONs nanoplatform for dual-modality MRI and NIR FI. ^D^ANG could specifically target the low-density lipoprotein receptor Protein 1 (LRP1) that was highly expressed on brain capillary ECs and GBM, while Cy7 enabled intraoperative real-time FI for locating GBM. ^D^ANG/Cy7-SPIONs with active targeting capability showed significant contrast enhancement effect for MRI as compared to that of Cy7-SPIONs probe. This demonstrates that the ^D^ANG represents an effectively and specifically targeting ligand for GBM, which holds great potential for future clinic translation.

CMT is capable of transporting nutrients, vitamins and hormones to the brain, and the transporters used are highly stereospecific for their substrates [39]. Substrates can bind to carrier proteins on one side of the cell membrane, generating an allosteric effect that moves the combined substrate to the other side of the membrane (Fig. 2d) [40]. A large number of carrier proteins are expressed on BBB, such as l-type amino acid transporter (LAT1), glucose transporter (GLUT1), cationic amino acid transporter (CAT1), choline transporter (ChT) and sodium-coupled glucose transporters (SGLTs), etc. [41]. For example, Li et al. constructed a choline derivative (CD)-modified DTPA-Gd, which had higher affinity than choline chloride for targeting both BBB ChT and GBM ChT, leading to a higher concentration in GBM than that of CD-free one. As a result, this dual-targeting nanoprobe could precisely detect the GBM even with the intact BBB [42].

The surfaces of brain capillary ECs are negatively charged under physiological pH conditions [43]. Based on this feature, another transport pathway for macromolecules crossing the BBB is AMT. It utilizes the electrostatic interaction between a positively charged substrate with negatively charged ECs, forming a vesicle for endocytosis (Fig. 2e) [44]. For example, the cell-penetrating peptides contain a highly alkaline amino acid sequence that imparts a positive charge on the peptide, and thus molecules labelled with cell-penetrating peptides are able to cross the BBB [45]. According to the report, AMT possesses a lower binding affinity but a higher transport saturation concentration in contrast to RMT [46].

Cell-mediated transcytosis has gained increasing attention over the past years. Multiple cell types including neural stem cells (NSCs), mesenchymal stem cells (MSCs), erythrocytes, platelets and tumor cells are explored as drug delivery systems [47–50]. The principle of drug delivery by erythrocytes is based on the unique feature of reversible opening under hypoosmotic conditions to encapsulate exogenous substances when the membrane pores are re-closed. However, the disadvantage is that erythrocytes cannot cross the endothelial barrier [51]. In contrast, NSCs [49], MSCs [52], platelets [53], macrophages [54], neutrophils [55] and tumor cells [47] exhibit intrinsic tumor-homing capacity (Fig. 2f), enabling them to deliver theranostic drugs to the brain tumor site [50]. For example, a Pt/MnO_2_@PVCL NGs nanoplatform with macrophage membranes as carriers to bear MnO_2_ and cisplatin (Pt) was designed for MRI-guided chemotherapy/chemodynamic therapy (CDT) of orthotopic GBM. Importantly, the macrophage membrane coating not only contributes to a high drug loading capacity but also allows hybrid NGs to have a longer circulation time and achieve high efficiency to cross the BBB. This led to very high drug concentration in the brain tumors, and significantly enhanced diagnostic and therapeutic outcomes [56].

BBB disruption-enhanced transport involves osmotic disruption and microbubbles (MBs)-induced BBB opening under US stimulation. The osmotic BBB disruption as a strategy often utilizes hypertonic mannitol solution to damage the TJs and cause ECs contraction, thereby opening the BBB [57]. However, the compromised BBB allows some large-molecules and harmful substances to enter the brain and affect the normal function of the central nervous system [58]. When exposure to low-energy focused US (FUS), MBs tend to explode to locally open the BBB (Fig. 2g) [59]. US-based techniques can reversibly open the BBB but the collapse or explosion of MBs during FUS irradiation is not easy to control and sometimes may damage the ECs [60, 61]. After opening BBB, the loaded drugs still rely on free diffusion to passively cross the BBB. With the help of FUS and magnetic targeting (MT), therapeutic MNPs were demonstrated to efficiently cross the BBB and reach to the magnetic target site, leading to the high local drug concentration. On the other hand, the MRI of the MNPs could be used to monitor and quantify the distribution in vivo, which further guided the conduction of therapeutic treatment (Fig. 2h) [62].

## Contrast agents for MRI and multimodal imaging modalities

In this section, we list the currently reported MRI and MRI-based contrast agents for GBM diagnosis. These contrast agents are summarized mainly from the following perspectives, including the constituent materials, targeting moieties, tumor models and imaging modalities (Table 1).

**Table 1:** Contrast agents for MRI and multimodal imaging modalities

Materials	Targeting moiety	Tumor model	Imaging method	References
Fe_3_O_4_ (MNP)	MT	Orthotopic C6 mice model	MRI(T2)	[62]
Lf-SPION	Lf	Orthotopic C6 mice model	MRI(T2)	[63]
HPF-NSCs	NSCs	Orthotopic U251T.eGFP.ffluc mice model	MRI(T2)	[64]
NPCP-BG-CTX	CTX and CED	Orthotopic GBM6-luc mice model	MRI(T2)	[65]
MGMSPID	Interleukin-13	Orthotopic U251 mice model	MRI(T2)	[66]
M-HFn	HFn	Orthotopic U87MG mice model	MRI(T2)	[67]
SPION-EGF	EGF	Orthotopic C6 mice model	MRI(T2)	[68]
Rhodamine-Mfls	MT	Orthotopic U87MG mice model	MRI(T2)	[69]
SPION-Hsp70	Hsp70	Orthotopic 9 L mice model	MRI(T2)	[70]
SD-MD	MT	Orthotopic C6 mice model	MRI(T2)	[71]
CARD-B6	B6	Orthotopic U87MG mice model	MRI(T2)	[72]
RGD-magnetosomes	RGD	Orthotopic U87MG mice model	MRI(T2)	[73]
CLIO-ICT	ICT2588	Orthotopic pcGBM39 mice model	MRI(T2)	[74]
ND-MMSNS	Neutrophils	Orthotopic U87-Luc/C6-Luc mice model	MRI(T2)	[75]
RGE-Exo-SPION/Cur	RGE	Orthotopic U251 mice model	MRI(T2)	[76]
NPCP-CTX	CTX and CED	Orthotopic GBM6 mice model	MRI(T2)	[77]
Ang-LiB(T + AN@siTGF-β)	Ang	Orthotopic GL261 mice model	MRI(T2)	[78]
IUdR/NGO/SPION/PLGA	MT	Orthotopic C6 mice model	MRI(T2)	[79]
I6P7-SPIO	I6P7	Orthotopic U87MG mice model	MRI(T2)	[80]
USPIO-PEG-tLyP-1	tLyP-1	Orthotopic U87MG mice model	MRI(T2)	[81]
PTPu-IO	PTPu	Orthotopic U87MG mice model	MRI(T2)	[82]
GrB-SPION	GrB	Orthotopic C6 mice model	MRI(T2)	[83]
NP-MTX-CTX	CTX	Subcutaneous 9 L mice model	MRI(T2)	[84]
NP-PEG-CTX	CTX	Subcutaneous 9 L mice model	MRI(T2)	[85]
MPGNPs	–	Subcutaneous C6 mice model	MRI(T2)	[86]
Fe_3_O_4_@Au-C225	C225	Subcutaneous U251 mice model	MRI(T2)	[87]
Gd-DTPA-DGLs-PEG-CTX	CTX	Orthotopic C6 mice model	MRI(T1)	[88]
DPC-DTPA-Gd	CD	Orthotopic U87MG mice model	MRI(T1)	[42]
Gd-NGO/Let-7 g/EPI	–	Orthotopic U87MG mice model	MRI(T1)	[89]
Au@DTDTPA-Gd	–	Orthotopic 9LGS mice model	MRI(T1)	[90]
Gd_3_N@C_80_(OH)_x_(NH_2_)_y_((amino-1))	Interleukin-13	Orthotopic U251 mice model	MRI(T1)	[91]
iRPPA@TMZ/MnO	iRGD	Orthotopic C6 mice model	MRI(T1)	[92]
Den-RGD-Reg + Gd^3+^-DTPA	RGD and Regadenoson	Orthotopic U87MG mice model	MRI(T1)	[93]
NaGdF4-TAT-labeled T cell	T cell	Orthotopic GL261 mice model	MRI(T1)	[94]
HA-MnO_2_	HA	Orthotopic C6 mice model	MRI(T1)	[95]
CPP-2	Ang	Orthotopic C6 mice model	MRI(T1)	[96]
MnO_2_@Tf-ppIX	Tf	Orthotopic C6 mice model	MRI(T1)	[37]
AGuIX@PS@KDKPPR	KDKPPR	Orthotopic U87MG mice model	MRI(T1)	[97]
Fe_3_O_4_-ANG	ANG	Orthotopic U87L mice model	MRI(T1)	[98]
M-CSTD.NHAC/Cu(II)	RGD and DER	Orthotopic C6 mice model	MRI(T1)	[99]
Pt/MnO_2_@PVCL NGs	Macrophage membrane	Orthotopic C6 mice model	MRI(T1)	[56]
HB-POEGMA-cRGD-Gd	cRGD	Subcutaneous U87MG mice model	MRI(T1)	[100]
rUCNPs@HSA(Ce6-Mn)-RGD	RGD	Subcutaneous U87MG mice model	MRI(T1)	[101]
Mn-ZIF-8/5-Fu	–	Subcutaneous U87MG mice model	MRI(T1)	[102]
Cu_2_(OH)PO_4_@PAA	–	Subcutaneous U251 mice model	MRI(T1)	[103]
PFC-labeled CAR T	CAR T	Subcutaneous U87-EGFRvIII-Luc mice model	^19^F MRI	[104]
TAT-PFC- labeled CAR T	CAR T	Subcutaneous U87-EGFRvIII-Luc mice model	^19^F MRI	[105]
G5-SA-D-Ac	CED	Orthotopic U87MG mice model	CEST-MRI	[106]
YbHPDO3A	–	Orthotopic U87MG mice model	CEST-MRI	[107]
Fe_0.6_Mn_0.4_O	–	Orthotopic U87MG mice model	MRI(T1/T2)	[108]
Fe-NCP	–	Orthotopic GL261 mice model	MRI(T1/T2)	[109]
Mn-NEB + BSA	–	Orthotopic U87MG mice model	MRI(T1/T2)	[110]
NP-S-S-PEP	RGD	Orthotopic U87MG mice model	MRI(T1/T2)	[111]
Fe_3_O_4_@SiO_2_@mSiO_2_/DOX-(Gd-DTPA)-PEG-RGE	RGE	Subcutaneous U87MG mice model	MRI(T1/T2)	[112]
D@HMON@FG@R2	RGD	Subcutaneous U87MG mice model	MRI(T1/T2)	[113]
POP/DCM@P-Mn-SPIO	–	Orthotopic 12FLR mice model	TMRET(T1/T2)	[114]
PFOB	RGD	Orthotopic U87MG mice model	^19^ F MRI/FI	[115]
Au-AZ/Au-AK	ANG	Orthotopic U87MG mice model	MRI(T1)/Raman	[116]
Cy5.5-Lf-MPNA	Lf	Orthotopic C6 mice model	MRI(T2)/FI	[117]
FluoroMags	–	Orthotopic GBM-NSs mice model	MRI(T2)/FI	[118]
QSC-Lip	MT	Orthotopic C6 mice model	MRI(T2)/FI	[119]
SPIO@DSPE-PEG/DOX/ICG	–	Orthotopic C6 mice model	MRI(T2)/FI	[120]
BFNP	–	Subcutaneous C6 mice model	MRI(T2)/FI	[121]
ICG-SPIO	–	Subcutaneous U251 mice model	MRI(T2)/PAI	[122]
Tb-doped MnCO_3_	–	Orthotopic C6 mice model	MRI(T1)/photoluminescence	[123]
CTX-NaGdF_4_:Ho^3+^	CTX	Orthotopic C6 mice model	MRI(T1)/FI	[124]
P/Gd-DTPA/cetuximab/MsTfR-mAb/Alexa-680	cetuximab/MsTfR-mAb	Orthotopic EGFR^+^U87MG mice model	MRI(T1)/FI	[125]
MnO	–	Orthotopic C6 mice model	MRI(T1)/FI	[126]
NCDDG	–	Orthotopic U87MG mice model	MRI(T1)/FI	[127]
Gd-Ag_2_S	–	Orthotopic U87MG mice model	MRI(T1)/NIR-II FI	[128]
CH4T@MOF-PEG-AE	AE105	Orthotopic U87MG mice model	MRI(T2)/NIR-II FI	[129]
Den RGD-Angio	RGD	Orthotopic U87MG mice model	MRI(T1)/NIR FI	[130]
Gd/MnCO_3_-PEG-Cy5.5-FA	FA	Orthotopic C6 mice model	MRI(T1)/NIR FI	[131]
MnO-PEG-Cy55	–	Orthotopic C6 mice model	MRI(T1)/NIR FI	[132]
ICG-FA-PPC	FA	Subcutaneous U87MG mice model	MRI(T1)/NIR FI	[133]
Cy5.5-Lf-SPIO	Lf	Orthotopic C6 mice model	MRI(T2)/NIR FI	[134]
Cy5.5-Fe_3_O_4_-PEG-RGD-FA	RGD and FA	Orthotopic C6 mice model	MRI(T2)/NIR FI	[135]
^D^ANG/Cy7-SPIONs	^D^ANG	Orthotopic Luc-U87MG mice model	MRI(T2)/NIR FI	[38]
NPC-Cy5.5	CTX	9 L cell	MRI(T2)/NIR FI	[136]
^64^Cu-DOTA-IO-RGD	RGD	Subcutaneous U87MG mice model	MRI(T2)/PET	[137]
Gd@C82-Ala-PEG-cRGD-(NOTA-^64^Cu or ^89^Zr)	cRGD	Subcutaneous U87MG mice model	MRI(T1)/PET	[138]
^64^Cu-cRGD-SPIO	RGD	Subcutaneous U87MG mice model	MRI(T2)/PET	[139]
^125^I-RGD-PEG-MNPs	RGD	Subcutaneous U87MG mice model	MRI(T2)/SPECT	[140]
RGD-Au-Mn DENPs	RGD	Orthotopic C6 mice model	MRI(T1)/CT	[141]
MPR	–	Orthotopic eGFP^+^ U87MG mice model	MRI(T1)/PAI/Raman	[142]
MSC-HA-MSNs-Gd^3+^-^64^Cu-ZW800	MSC	Orthotopic U87MG mice model	MRI(T1)/PET/NIR	[143]
HALF-cRGD	cRGD	Orthotopic C6 mice model	MRI(T2)/PAI/FI	[144]
cRGD-CM-CPIO	cRGD	Orthotopic C6 mice model	MRI(T2)/PAI/FI	[145]
Au@MIL-88(Fe)	–	Orthotopic U87MG mice model	MRI(T2)/CT/PAI	[146]
Fe_3_O_4_@Au	α_v_β_3_ mAb	U87MG cell	MRI(T2)/CT/PAI	[147]
Gd-PEG-Bi	–	Subcutaneous U87MG mice model	MRI(T1)/CT/PAI	[148]
^64^Cu-Fe-RGD-PEG-MNP	RGD	Subcutaneous U87MG mice model	MRI(T1)/PET/PAI	[149]

## Magnetic cores for MRI

In this section, we classify the available magnetic cores for MRI into five major categories including iron oxide NPs, Gd-based NPs, manganese (Mn)-based NPs, ^19^F MRI and copper (Cu)-based NPs. With the rapid development of nanomedicine technology, surface modification on the contrast agents can decrease their toxicity and increase their biocompatibility, especially endow them with the abilities of BBB crossing and tumor targeting as well as therapeutic units.

### Iron oxide NPs

**Fig. 3:**
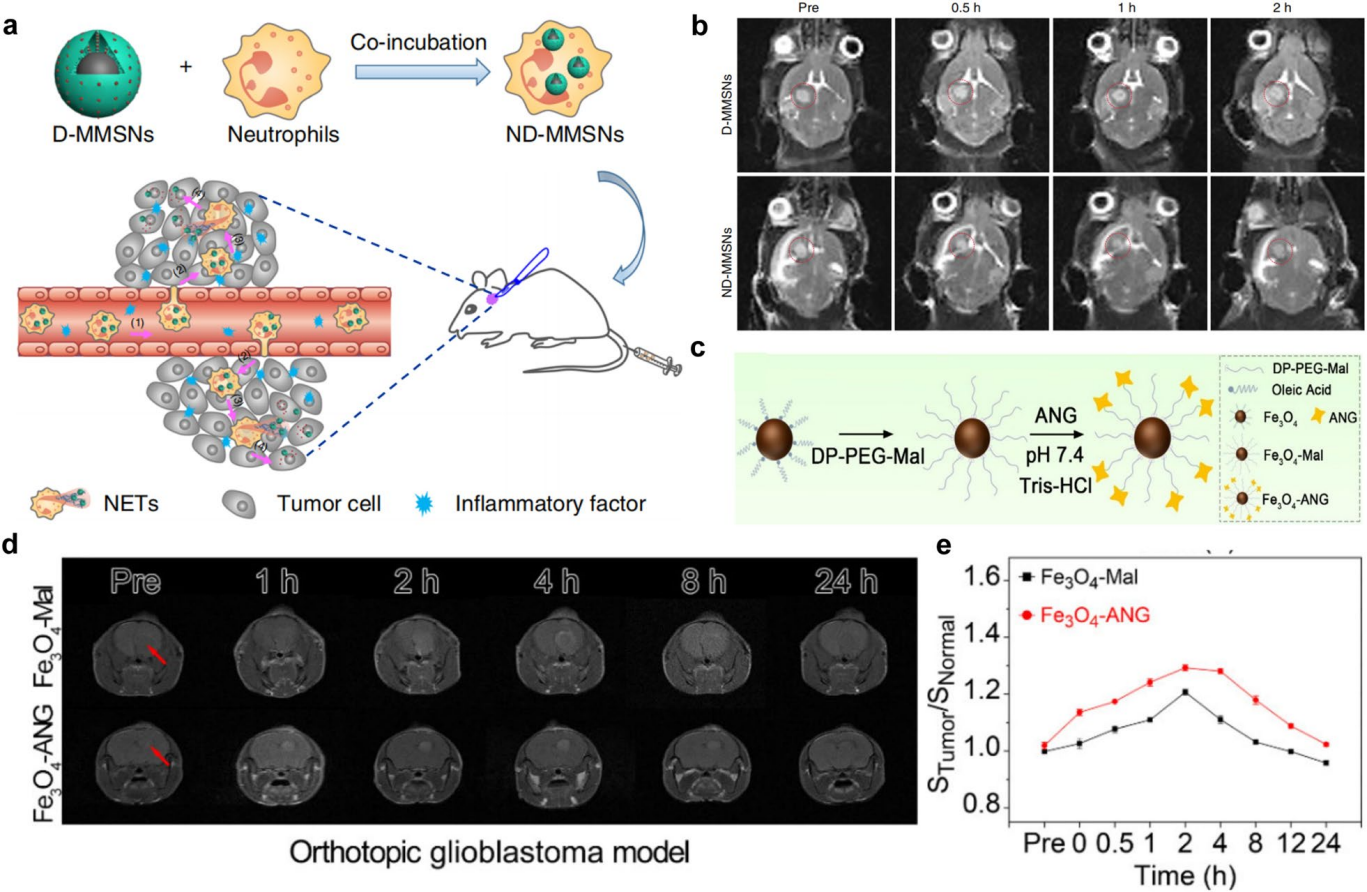
Iron oxide for MRI. **a** Schematic representation of ND-MMSNs synthesized and targeted to postoperative GBM. **b** In vivo T2WI of postoperative GBM model before and after intravenous injection of D-MMSNs and ND-MMSNs. (adapted from [75] under Creative Commons CC BY license). **c** Schematic diagram of the synthesis of Fe_3_O_4_-Mal and Fe_3_O_4_-ANG nanoprobe. **d** MRI of the orthotopic GBM model within 24 h after injection of Fe_3_O_4_-Mal or Fe_3_O_4_-ANG NPs. **e** Tumor signal trends for the orthotopic GBM model after injection of Fe_3_O_4_-Mal or Fe_3_O_4_-ANG NPs. Adapted from [98] under Creative Commons CC BY license

T2WI is a basic MRI sequence that shows differences in the T2 relaxation times of the tissue. For example, tumor necrosis and peritumor edema usually have higher water concentrations and show bright signals in T2WI because the long relaxation times of water molecules [150]. In recent years, iron oxide NPs have received increasing attention and widely been used as T2-negative MRI contrast agents, due to their strong capability to shorten the T2 relaxation time in the adjacent regions. It should be noticed that the enrichment of iron oxide NPs in disease tissue generally results in a reduced MRI signal in T2WI as a dark signal, which forms strong contrast opposite to normal tissues [151]. According to an outer-sphere theory [152], the R_2_/R_1_ ratio increases as the particle size increases, so smaller particles are better T1 shortening agents than larger ones. Therefore, SPIONs with larger size are developed as T2WI contrast agents, while the new generation of ultrasmall superparamagnetic iron oxide NPs (USPIONs) with a diameter less than 10 nm show typical T1-enhancing characteristics [152]. Importantly, Iron oxide can be metabolized by macrophages in the mononuclear phagocytic system and stored as iron to synthesize hemoglobin, which contributes to its good biocompatibility and great promise in translation from bench to bed-side [153]. However, it has been found that the relatively large size of SPIONs would cause easy and rapid clearance by phagocytes, hampering their further application for molecular imaging [154]. Besides, the T2WI is easily confused with hemorrhage and calcification, etc. Therefore, USPIONs become advantageous because they can shorten the T1 relaxation time of water protons and act as a T1-positive contrast agents [155]. Wang et al. [98] modified 3.3 nm-sized ultra-small Fe_3_O_4_ with Angiopep-2 (ANG) using DP-PEG-Mal as a linker (Fig. 3c), in which the ANG could target the highly expressed LRP1 on BBB and GBM. The gradual enhancement of MRI contrast was observed on T1WI after injection of Fe_3_O_4_-Mal or Fe_3_O_4_-ANG into mice within 24 h, and reached the maximum at 4 h and 2 h, respectively (Fig. 3d). Due to the active targeting of ANG, Fe_3_O_4_-ANG NPs exhibited higher contrast increment than Fe_3_O_4_-MAL NPs (Fig. 3e). The R_1_ of Fe_3_O_4_-ANG was calculated to be 7.45 mM^− 1^s^− 1^, which was higher than that of the Gd-DTPA (R_1_ = 3.32 mM^− 1^s^− 1^). In addition, it has been demonstrated that the obtained nanoprobe possessed good biocompatibility. Therefore, Fe_3_O_4_-ANG nanoprobe with high R_1_ is a promising T1 contrast agent for GBM diagnosis.

In order to target GBM, recombinant human epidermal growth factor (EGF) [68], EGFR monoclonal antibody (McAb) cetuximab (C225) [87], CTX [85], heat shock protein Hsp70 [70], hydrophilic peptide I6P7 [80], polypeptide tLyP-1 [81], and the serine protease Granzyme B (GrB) [83] have also been reported to modify iron oxide NPs. These nanoplatforms can carry chemotherapeutic agents such as potent vascular disrupting agent (ICT) [74], curcumin (Cur) [76] and doxorubicin (Dox) [120] to realize MRI-guided treatment of GBM. Specifically, Wu et al. [75] reported the inflammation-activatable engineered neutrophils *via* internalization of DOX-loaded Fe_3_O_4_/mesoporous silica core-shell NPs (ND-MMSNs) and then investigated the diagnostic and therapeutic effects on an incompletely resected GBM model. Due to the phagocytic capacity of neutrophils, it can engulf D-MMSNs to obtain a smart bionic nanotheranostics ND-MMSNs, which could target the areas of postoperative GBM (Fig. 3a). The tumor homing ability of neutrophils was monitored on T2WI. Compared with the D-MMSNs, the ND-MMSNs group exhibited stronger negative contrast enhancement in the postoperative GBM area (Fig. 3b). Meanwhile, postoperative mice treated with ND-MMSN showed significantly improved survival rate and delayed recurrence. In this study, ND-MMSNs exhibited strong cell tracking capability which offers an efficient paradigm for diagnosing and guiding treatment of residual tumors.

**Fig. 4:**
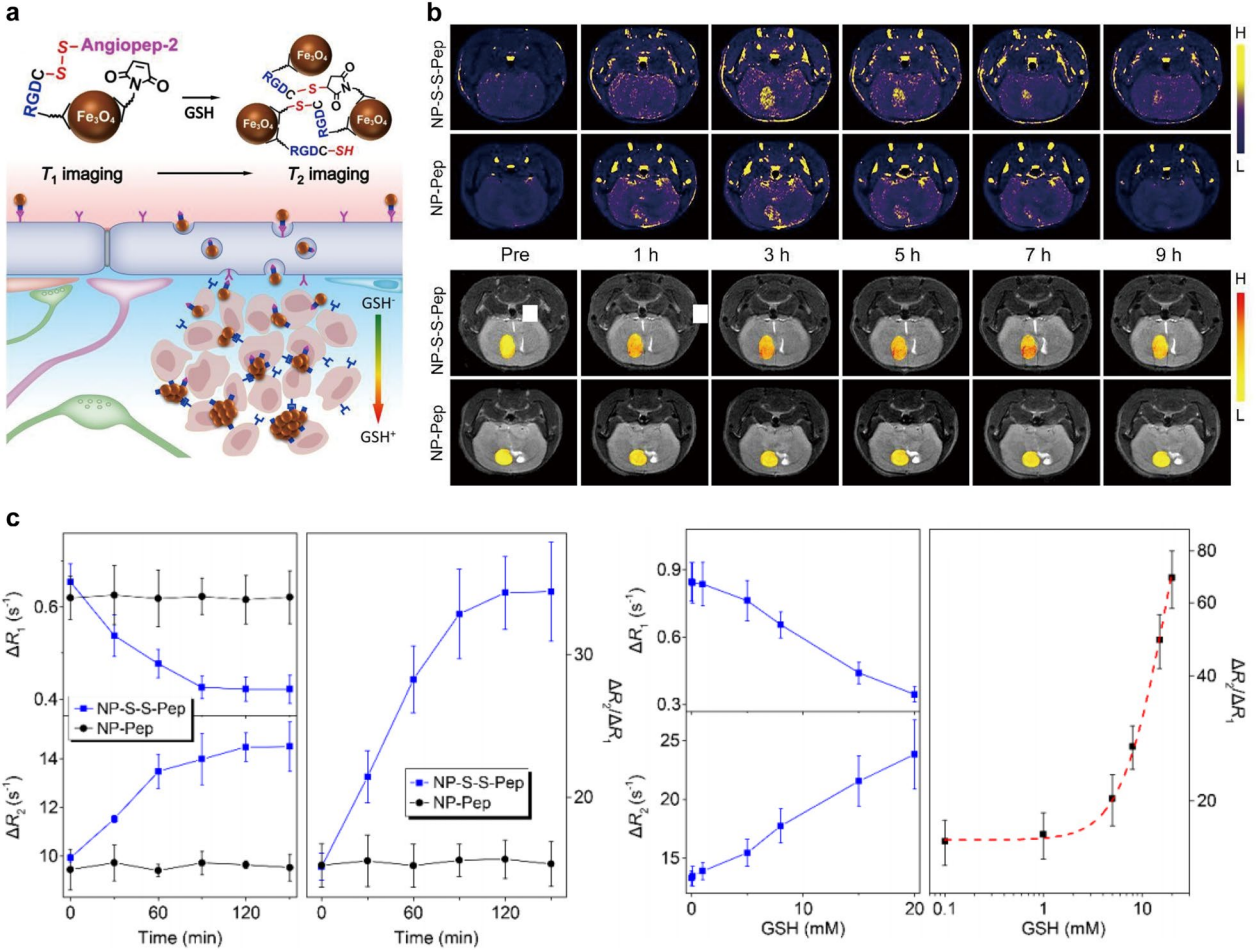
Iron oxide NPs for T1WI and T2WI. **a** Schematic representation of the molecular mechanism of GSH-induced aggregation of NP-S-S-Pep probes. **b** T1WI and T2WI of mice bearing orthotopic GBM model acquired before and at different time points after the intravenous injections of NP-S-S-Pep and NP-Pep probes, respectively. **c** Time evolution of ΔR_1_, ΔR_2_ and ΔR_2_/ΔR_1_ of NP-S-S-PEP and NP-PEP probes at different concentrations of GSH. Reprinted with permission from [111]; copyright (2021) John Wiley and Sons, Inc.

In addition, the design of TME-responsive MRI contrast agents has been regarded as the research hotpot in recent years, which could significantly increase the imaging sensitivity and enhance the signal-to-background ratio. For example, Zhang et al. [111] developed a glutathione (GSH)-responsive MRI probe based on Fe_3_O_4_ NPs, which could induce aggregation when encountering the high GSH concentrations in the TME (Fig. 4a). Both T1WI and T2WI of GBM were performed on a mouse orthotopic brain tumor model to establish a quantitative correlation between local GSH level and MRI signal intensity (Fig. 4b). These interlocked responses effectively increased the GSH detection sensitivity, and a mathematic model was established with the help of theoretical analysis to quantitatively mapping the GSH in GBM through MRI. By subtracting the R_1_ and R_2_ of intrinsic solvent, the temporal variations of ΔR_1_, ΔR_2_ and ΔR_2_/ΔR_1_ are shown in Fig. 4c. R_1_ decreased with increasing GSH concentration, while R_2_ showed the opposite trend. Apparently, the GSH-induced variation can be better reflected by ΔR_2_/ΔR_1_. This research provides a practical method for quantitative mapping of tumor-specific biomarkers in vivo. There is another TME-responsive nanoplatform reported by Jiang et al. [117], they conjugated Cy5.5-labled lactoferrin (Lf) with pH/temperature-sensitive magnetic nanogels to synthesize pH-responsive Cy5.5-Lf-MPNA nanogels. Under physiological conditions, Cy5.5-Lf-MPNA nanogels were hydrophilic and exhibited prolonged blood circulation time. While in the acidic TME, they became hydrophobic and could be more easily accumulated in tumor tissues. Therefore, this probe actively targeted tumor with the assistance of Lf ligand, and performed efficient tumoral accumulation by the pH stimulus changes on hydrophily, leading to the high local probe concentration in tumors and strong image signals.

**Fig. 5:**
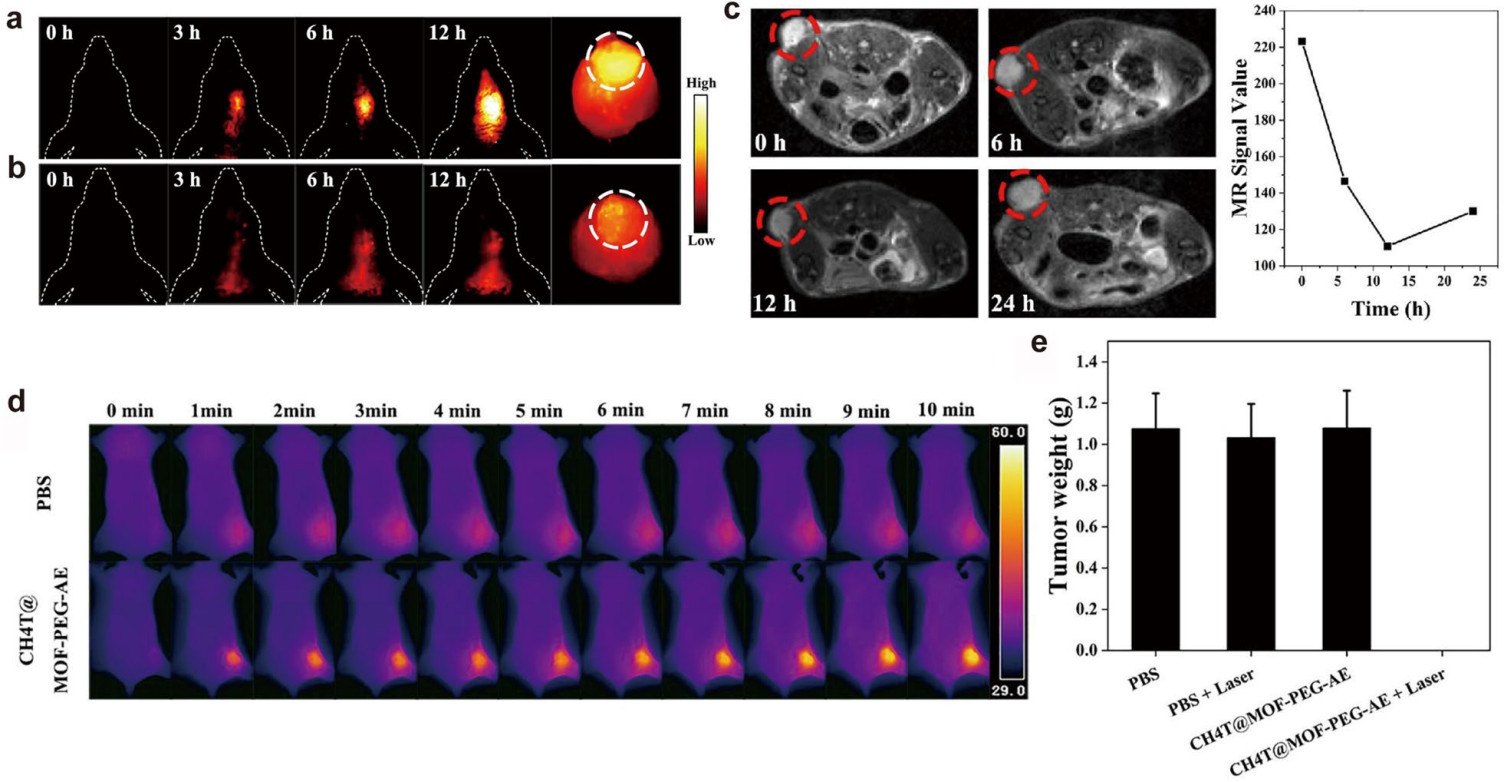
Iron oxide NPs for MRI ang NIR-II FI. NIR-II FI of the orthotopic GBM models after tail intravenous injection of **a** CH4T@MOF-PEG-AE or **b** CH4T@MOF-PEG-SCM. **c** T2WI and the corresponding MRI signal values before and after CH4T@MOF-PEG-AE treatment. **d** Thermal images of PBS group and CH4T@MOF-PEG-AE group in ten minutes. **e** Tumor weight in different treatment. Reprinted with permission from [129]; copyright (2021) Elsevier Ltd.

For dual-modal imaging of GBM, Xu et al. [119] encapsulated SPIONs, quantum dots (QDs) and cilengitide (CGT) in liposomes to form QSC-Lip for targeted GBM under MT and guided surgical resection by MRI/FI. Another multifunctional platform denoted as ^125^I-RGD-PEG-MNPs was developed to realize MRI/SPECT-guided photothermal therapy (PTT) of tumors in vivo [140]. Li and co-workers [129] assembled small-molecule NIR-II fluorophore (CH4T), Fe-based metal-organic framework (MOF), and tumor-targeting AE105 peptide into a CH4T@MOF-PEG-AE nanoprobe, which possessed a particle size of about 60 nm and an average hydrodynamic size of about 132.2 nm [polydispersity index (PDI) = 0.166)] Compared to the passive-targeted CH4T@MOF-PEG-SCM, the CH4T@MOF-PEG-AE exhibited stronger NIR-II fluorescence signal (Fig. 5a and b). The tumor area displayed a significant dark signal on T2WI after intravenous injection of CH4T@MOF-PEG-AE and the contrast reached the darkest at 12 h post-injection (Fig. 5c). The in vivo photothermal effects were monitored by thermal imaging camera, and results indicated that the tumor area of nanoprobe administration group reached 50 °C after 5 min laser irradiation and eventually rose to 56 °C, while the PBS + laser group only showed a slight temperature increment (Fig. 5d). Therapeutic experiments revealed that the U87MG cells were significantly killed and the tumors were eliminated without recurrence in the CH4T@MOF-PEG-AE plus laser group (Fig. 5e). Besides, CH4T@MOF-PEG-AE could guide surgical resection for deep GBM by NIR-II imaging with high sensitivity and accuracy, possessing great potential for GBM theranostics.

Advanced trimodal molecular imaging nanoprobes have also been studied in preclinical and clinical studies. For example, Duan et al. [144] integrated NIR molecules (TC1), cRGD peptides and ultrasmall iron oxide NPs (UIONPs) to prepare HALF-cRGD nanocomposites by a modified nanoprecipitation method, in which the UIONPs were confined to half of the nanosphere. This unique nanostructure physically separated TC1 and UIONPs with the ability to mitigate fluorescence quenching, thereby preserving the good performance of both FI, PAI and MRI. The synthesized multimodal imaging nanocomposite showed good imaging sensitivity on early-stage GBM, via integrating the merits of each imaging modality. Shang et al. [146] developed the core-shell gold nanorod@nanoscale metal-organic frameworks (NMOFs) nanoprobe using a microemulsion approach. The inner gold nanorod core possessed CT-enhanced and PAI optical properties, while the NMOFs shell severed as T2WI contrast agent. Interestingly, organic linkers in NMOFs can be easily customized to allow facile manipulation on biophysical properties of NMOFs for different biomedical applications such as drug delivery and imaging, promoting their potential in preclinic and clinic translation.

### Gd-based NPs

Gd-based contrast agents (GBCAs) are FDA-approved MRI contrast agents [156]. It is known that GBCAs shorten the T1 relaxation time of protons, which contributes to the fast imaging speed and less interference from motion generated artifacts [157]. Under normal conditions, GBCAs cannot cross the BBB. However, due to their small size, they could extravasate from the blood into the brain tissue even if the BBB is partially damaged. Therefore, intravenous injection of GBCAs can enhance the contrast between the tumor and normal brain tissue [158]. Notably, Gd chelates are cleared through the renal in vivo, while the excretion is dependent on the size of GBCAs [159, 160]. Unfortunately, the disadvantage of GBCAs is that they can release free Gd^3+^ to cause nephrogenic systemic fibrosis in patients with renal dysfunction [161]. American College of Radiology guidelines recommend against the use of any Gd in patients with acute kidney injury or glomerular filtration rate less than 40 mL/min/1.73 m [162]. Recently, it has been discovered that a portion of the injected GBCA remains in the body for a long time. Gd can be deposited in the brain even in patients without renal dysfunction. Repeated use of GBCAs would result in the accumulation of residual Gd^3+^ to detected levels by MRI or other approaches [163]. Despite to the above-mentioned disadvantages of GBCAs, they are still widely used as MRI contrast agents in clinical practice and exhibit an overall safety profile [164]. Low molecular weight Gd chelates such as Gd-DTPA and Gd-DOTA have been clinically used, but their rapid renal clearance causes insufficient concentration at the tumor site and deficient image contrast, limiting the further application for brain tumor imaging [165]. In contrast to the small molecular Gd chelates, encapsulation of Gd-chelates into nanocarriers such as liposomes, mesoporous silica, polymers, and plasmonic NPs, could inhibit the uncontrolled release profile for free Gd, which significantly contributes to the lower toxicity and enhanced circulation time [166]. Importantly, the chemical structure, material and size of Gd NPs would affect their metabolic pathway *in vivo.* Therefore, it needs to systematically further investigate whether the Gd NPs can overcome the before-mentioned drawbacks of GBCAs [167]. Up to now, a number of Gd-based NPs have been reported for brain tumor theranostics. For example, Yang et al. [89] constructed a Gd-NGO/Let-7 g/EPI nanoplatform using positively charged poly(amidoamine) dendrimer-grafted Gd-functionalized nanographene oxide (Gd-NGO) to adsorb anticancer drug epirubicin (EPI) and gene targeting agent Let-7 g miRNA. This NPs could inhibit cancer cell growth and simultaneously act as MRI contrast agent for tumor detection.

**Fig. 6:**
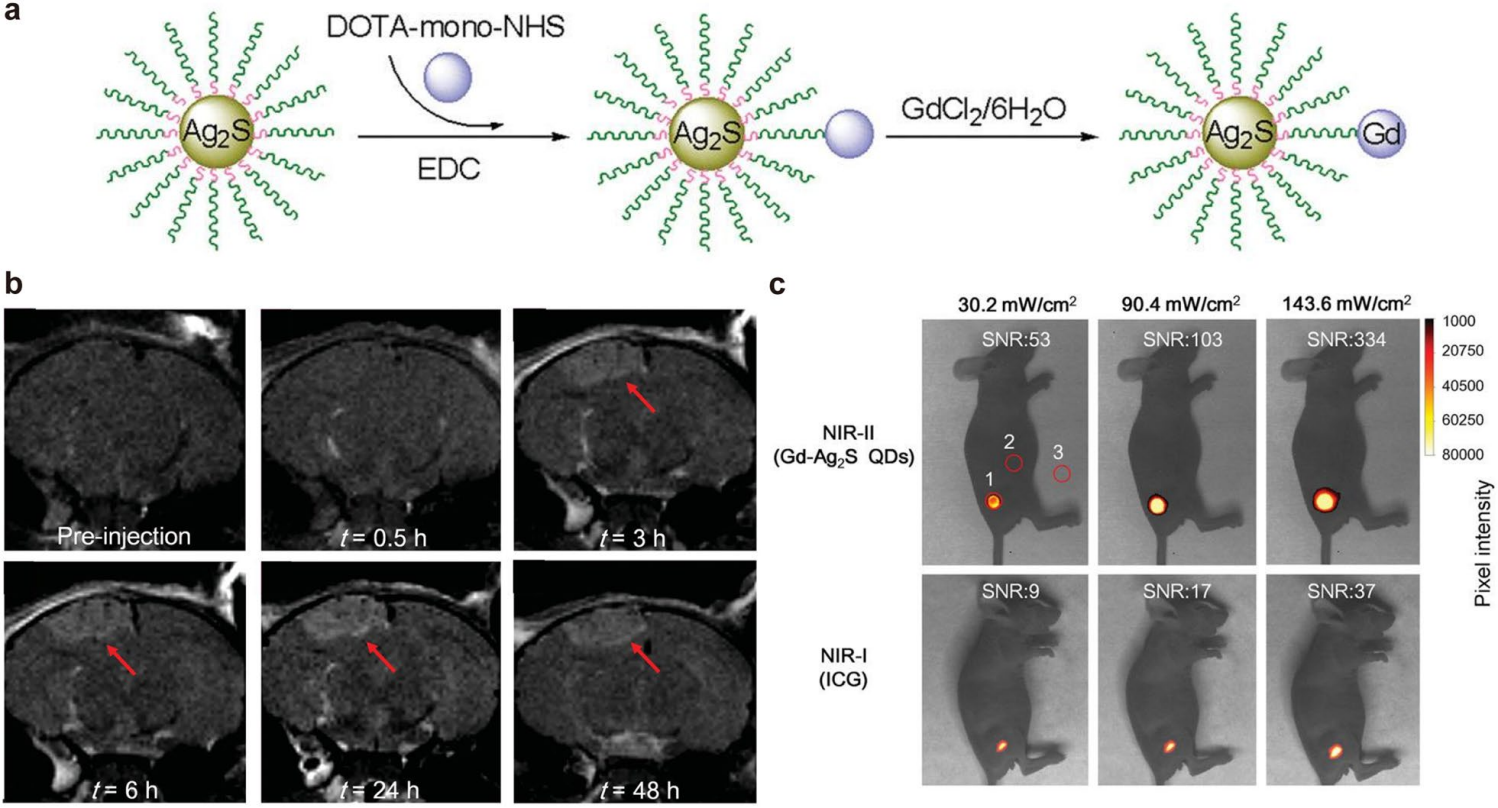
Gd-based NPs for MRI and NIR-II FI. **a** Schematic diagram of the synthesis of Gd-Ag_2_S nanoprobe. **b** MRI of Gd-Ag_2_S NPs before and after injection. **c** NIR-II FI of Gd-Ag_2_S nanoprobe and NIR-I FI of ICG at equimolar concentration as reference. Reprinted with permission from [128]; copyright (2015) John Wiley and Sons, Inc.

For dual-modal imaging, Li et al. [128] developed a uniform nanoprobe composed of Ag_2_S QDs and Gd complex (denoted as Gd-Ag_2_S) (Fig. 6a). The existence of Gd endowed the nanoprobe with ability of MRI for preoperative GBM diagnosis (Fig. 6b). In addition, compared with equivalent concentrations of indocyanine green (ICG), Gd-Ag_2_S QDs provided higher signal-to-noise ratio and can be for NIR-II FI-guided tumor resection intraoperatively (Fig. 6c), which indicates that dual MRI and NIR-II FI would greatly innovate the brain tumor diagnostics for pre- and intra-operative treatment. Another report, Patil et al. [125] developed a “MRI virtual biopsy” method. They designed a polymeric nano-imaging agent (NIA) consisted of Gd for MRI and Alexa-680 for FI. This NIA was able to cross the BBB by TfR antibody-targeted modification. In a model of double human brain tumors in mice mimicking brain metastasis, the NIA could be modified with specific antibodies for tumor targeting, such as trastuzumab for HER2^+^ breast cancer targeting or cetuximab for EGFR^+^ U87MG GBM targeting. Moreover, these specific antibodies have proved to inhibit tumor growth. Importantly, the above-mentioned technique allows to achieve real-time differentiation of tumor types, which is hard to achieve for biopsy.

**Fig. 7:**
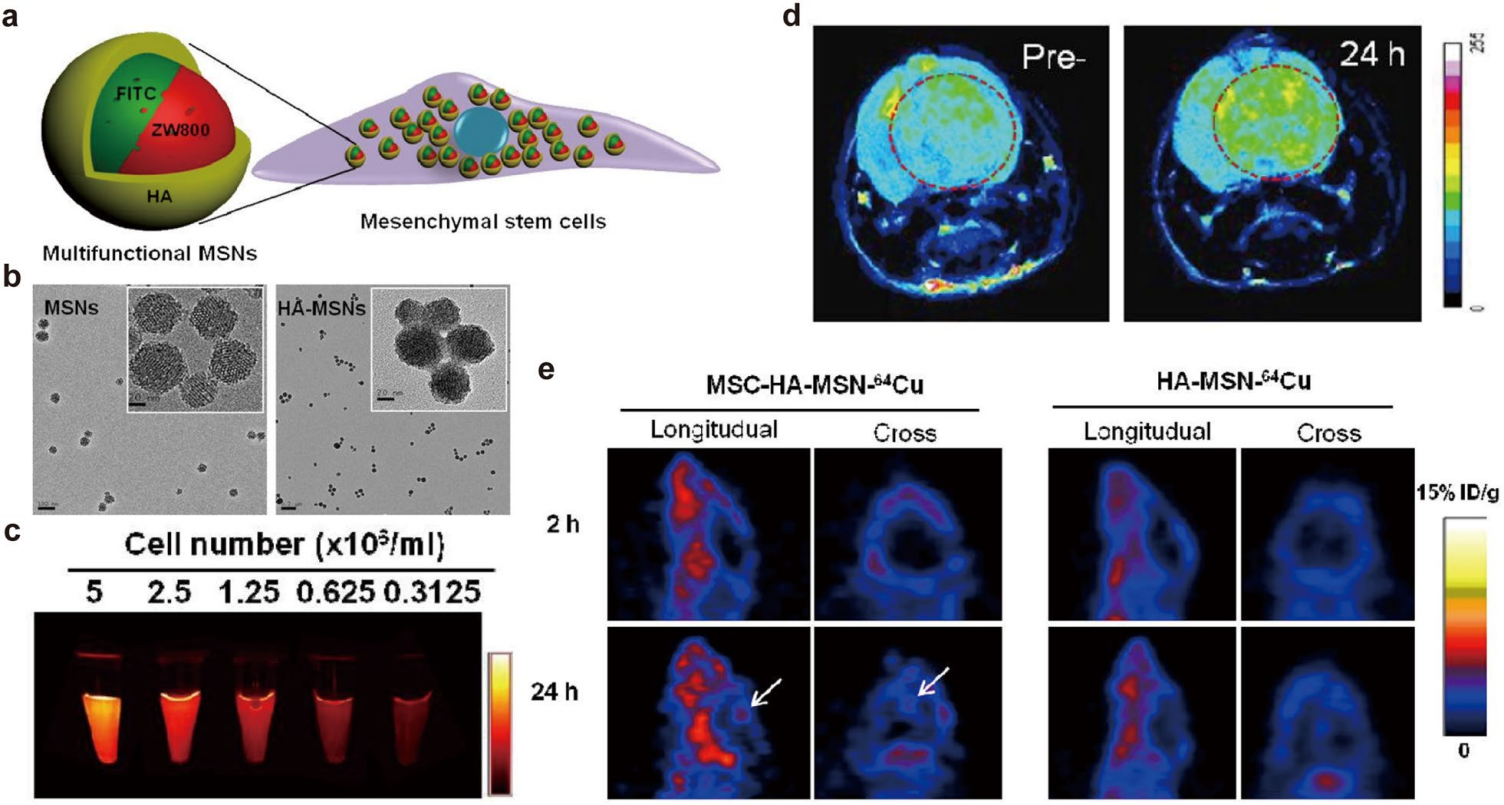
Gd-based NPs for triple-modal imaging. **a** Schematic of the structure of the MSC-platform. **b** TEM images of MSNs and HA-MSNs NPs. **c** The NIR fluorescence signal from ZW800 dye varied with an increase in MSCs concentration. **d** The increased T1 signal at the Orthotopic GBM mice model after MSC-platform administration for 24 h compared with pre-injection. **e** PET imaging of the MSC-platform and HA-MSN-^64^Cu. Reprinted with permission from [143]; copyright (2013) Elsevier Ltd.

For triple-modal imaging, Huang et al. [143] combined the MSCs and multifunctional mesoporous silica NPs (MSNs) with fluorescein isothiocyanate (FITC) and NIR dye ZW800 doped, hyaluronic acid (HA)-based polymer coated and Gd^3+^ and ^64^Cu labeled for NIR FI, MRI and PET imaging (Fig. 7a). The clear mesoporous structure of MSNs was observed by transmission electron microscope (TEM), but the pores were sealed after HA modification (Fig. 7b). The intensity of NIR imaging signal from ZW800 dye strengthened with the increase of MSCs concentration, indicating that NPs were integrated with MSCs (Fig. 7c). Compared with the pre-injection period, the T1 signal was significantly enhanced on T1WI after MSC-platform injection in the orthotopic GBM mice model (Fig. 7d). The tumor homing ability of MSCs was well confirmed in PET imaging, the signal at the tumor site of injected MSC-platform group was much stronger in contrast to the control (HA-MSN-^64^Cu group) (Fig. 7e). In this work, MRI could reveal the distribution of MSCs in tumor areas, while PET imaging is used to understand the dynamics of the MSC-platform, and optical imaging helps to monitor the interaction between MSNs and MSCs. It’s obviously that complementary imaging techniques could improve the tracking accuracy of MSC-platform in vivo.

### Mn-based NPs

Mn is a vital nutrient for intracellular activities and acts as a cofactor for various enzymes [168]. Paramagnetic Mn induces a strong reduction in T1 relaxation time of proton, which can be used as a contrast agent for T1WI [169]. However, free-form of Mn^2+^ is toxic and therefore Mn chelates such as Mn^2+^-based complexes and manganese oxide NPs (MONs) are commonly used to prevent premature release of metal ions and to enhance the T1 signal [170]. Due to the short circulation time of Mn^2+^-complexes and potential neurotoxicity of high doses of Mn^2+^, MONs with negligible toxicity and good T1-weighted contrast effects is regarded as a decent choice. Moreover, MONs can respond to the TME to alleviate tumor hypoxia and improve the therapeutic effect [171]. For example, Tan et al. [92] incorporated oleic acid-modified manganese oxide (MnO) and temozolomide (TMZ) into an arginine-glycine-aspartic acid (iRGD) containing polyethylene glycol-poly(2-(diisopropylamino)ethyl methacrylate micelle and yielded iRPPA@TMZ/MnO nanoplatform. The iRPPA@TMZ/MnO could specifically target the GBM tissues, in which MnO rapidly responded to the TME and generated Mn^2+^ and O_2_. This causes downregulated HIF-1α expression and alleviated tumor hypoxia, thereby increasing tumor sensitivity to Mn^2+^-induced CDT and TMZ-caused chemotherapy. As compared to conventional GBCAs or SPIONs, Mn-mediated MRI has advantages in clear visualization on the subatomic structure of the brain and its neuronal activity [172]. Importantly, intravenously injected Mn-chelates can be rapidly cleared by mixed renal and hepatobiliary pathway. This would reduce the unnecessary accumulation in vivo for Mn-chelates, which is of high significance for their clinic application [173].

**Fig. 8:**
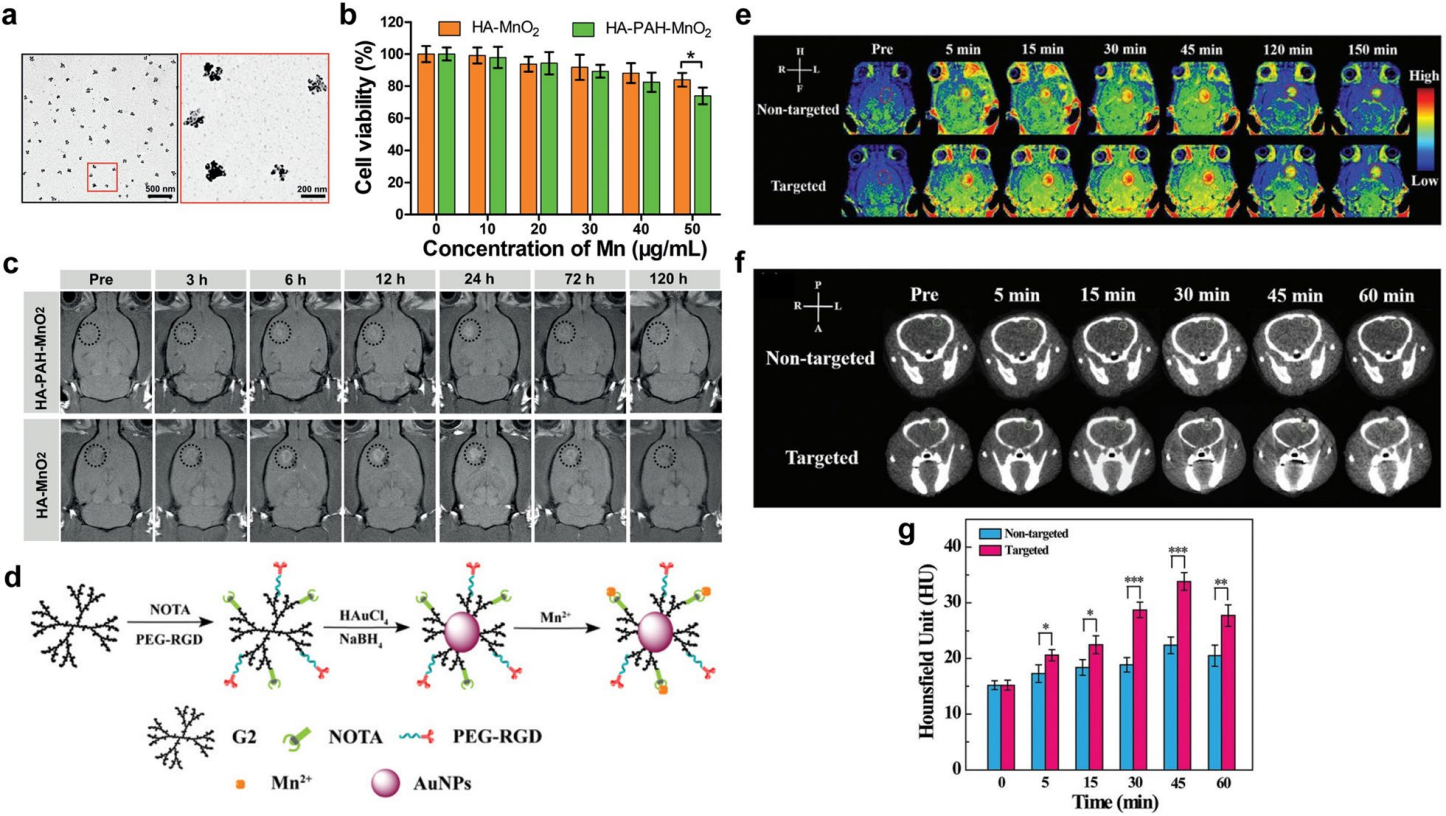
Mn-based contrast agents. **a** TEM images of HA-MnO_2_ NPs. **b** Cell viability of C6 glioma cells after incubation with HA-MnO_2_ NPs and HA-PAH-MnO_2_ NPs at varying Mn concentrations. **c** In vivo T1WI after intravenous injection of HA-PAH-MnO_2_ NPs and HA-MnO_2_ NPs [reprinted with permission from [95]; copyright (2019) John Wiley and Sons, Inc.]. **d** Schematic diagram of the synthesis of RGD-Au–Mn DENPs. **e** The T1WI of the C6 orthotopic glioma tumor before and after the non-targeted Au–Mn DENPs or targeted RGD-Au–Mn DENPs. The CT images (**f**) and quantitative CT values (**g**) of the C6 orthotopic glioma tumor before and after the non-targeted Au–Mn DENPs or targeted RGD-Au–Mn DENPs were intravenously injected, respectively [reprinted with permission from [141]; copyright (2019) Royal Society of Chemistry]

In addition, Fu et al. simply mixed sodium permanganate with HA aqueous solution to synthesize multifunctional HA-MnO_2_ NPs for brain tumor imaging. As shown in TEM images, the HA encapsulated MnO_2_ showed sphere-like morphology, while unassembled MnO_2_ formed individual clusters (Fig. 8a). HA-MnO_2_ NPs displayed lower cytotoxicity against C6 glioma cells compared to that of HA-PAH-MnO_2_ NPs, which were prepared by the conventional reduction reaction between cationic poly-(allylamine hydrochloride) (PAH) with potassium permanganate (Fig. 8b). Moreover, the T1WI signal intensities of tumor sites after the injection of HA-MnO_2_ NPs were significantly higher than those after the injection of HA-PAH-MnO_2_ NPs (Fig. 8c). This study successfully developed a MnO_2_-based nanoplatform formulated through one-step method for imaging and therapy of brain tumors [95].

For dual-modal imaging, Xu et al. [141] decorated Au NPs and Mn^2+^ on RGD peptide modified poly(amidoamine) (PAMAM) dendrimers of generation 2 (G2) to obtain RGD-Au-Mn DENPs nanoplatform (Fig. 8d). In contrast to Au-Mn DENPs, the RGD-Au-Mn DENPs with tumor targeting ability showed stronger T1WI signal on orthotopic C6 mice model (Fig. 8e). Moreover, the CT values in the targeted group were 1.5 times higher than those in the non-targeted group at the peak time point of 45 min (Fig. 8f and g). Importantly, RGD-Au-Mn DENPs possessed a high R_1_ relaxivity (9.88/mM/s) and as well as better CT imaging performance than iodine-based CT contrast agents. These results demonstrate that RGD-Au-Mn DENPs are prospective dual-modal MRI/CT imaging probes specifically for GBM. For dual channel MRI, Wang et al. [114] developed a disulfide crosslinked micelle (DCM)-encapsulated paramagnetic Mn^2+^ chelate (P–Mn) and SPIO nanoplatform (denoted as DCM@P-Mn-SPIO), which could be used in a new two-way magnetic resonance tuning (TMRET) nanotechnology with dual activation of T1 and T2 signals in response to GSH. Quenching behaviors of T1 and T2 MRI signals occurred when the TMRET pair was tightly locked within the micellar core. However, the signals recovered upon biological stimuli due to the increased distance between Mn^2+^ and SPION, which was controlled by the integrity of the micelles. This method was also feasible in other TME-responsive micellar nanostructures such as pH-responsive PEG_5000_-OH_8_-PPBA (POP, PPBA = porphyrin phenylboronic acid). Both T1 and T2 MRI signal intensities of intracranial tumors were significantly enhanced after injection of POP@P-Mn-SPIO. The experimental results showed that the quenching behavior of R_1_ and R_2_ of POP@P-Mn-SPIO could be recovered by the stimulation of acidic pH (5.0). In this report, TMRET nanotechnology with post-imaging processing and reconstruction method could be used to diagnose ultra-small intracranial tumors (less than 1 mm).

### ^19^F MRI

Similar to Traditional proton (^1^ H) MRI, ^19^F MRI produces imaging signals by detecting the magnetic field changes which are accompanied by that ^19^F atoms return from the excited state to the ground state after the withdrawal of the radio frequency pulse [174]. ^1^ H MRI is able to present abundant anatomical and pathophysiological information, but it shows limited capabilities to visualize key cells and biomolecules which tend to be rare [175]. ^19^F MRI has great potential for diagnostic molecular imaging through attachment of fluorinated molecules to targets for cell tracking and oxygen sensing. However, it is known there are the only trace amounts of fluorine in living organisms, which is far less than the threshold dosage to achieve clear pinpointing of target tissues. Therefore, exogenous ^19^F probes have been developed to bring in sharp contrast between target site and normal tissues. ^19^F MRI is less developed than ^1^H MRI, in part due to the lack of sensitive biocompatible probes [176].

There are some reports on ^19^F MRI probes for brain tumors detection. For example, Giraudeau et al. [115] modified perfluorooctylbromide (PFOB) NPs with RGD peptide and rhodamine to prepare RGD-functionalized PFOB NPs, which could target neovascularization in a mouse GBM model. ^19^F images of tumors obtained after RGD emulsion injection were larger than 3 mm, corresponding well to anatomical ^1^H images. Moreover, the ^19^F signal distribution was also visually correlated with hematoxylin and eosin (HE) staining image and rhodamine image, indicating that ^19^F image can map tumor angiogenic activity. PFOB demonstrated good potential for guidance of quantitative and qualitative angiogenesis on GBM. Chapelin et al. [104] constructed a perfluorocarbon (PFC) nanoemulsion imaging tracer probe that could label chimeric antigen receptor (CAR) T cells and measure the intracellular tumor cell pressure of oxygen (PO_2_) by ^19^F MRI in a murine immunotherapy model. The results showed that the PO_2_ temporal dynamics in tumor cells were consistent with significant tumor killing effects and CAR T cell infiltration. This probe provided insight into the function of effector cells and tumor response in cellular immunotherapy cancer models. It should be noticed that PFC is non-metabolizable in cells, but they could be removed in liver when the Kupffer cells take up the dead cells contain PFC [105].

### Cu-based NPs

**Fig. 9:**
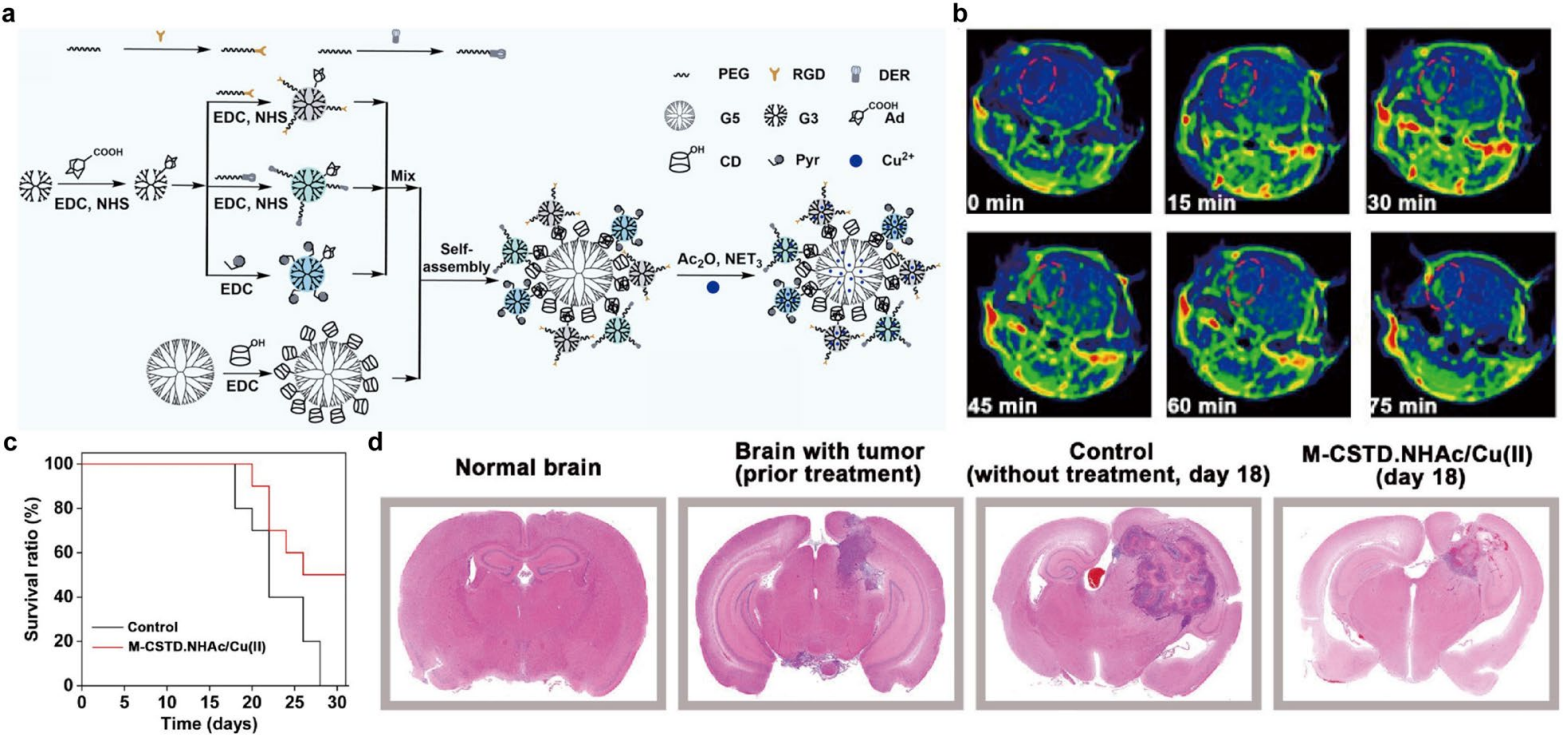
Cu for MRI. **a** Schematic diagram of composition of multifunctional M-CSTD.NHAc/Cu(II) complexes. **b** Representative in vivo T1WI of glioma after intravenous injection of the M-CSTD.NHAc/Cu(II) complexes. **c** Survival radio of mice treated with the M-CSTD/Cu(II) complexes or control. **d** HE staining of the brain tissues after different treatments. Reprinted with permission from [99]; copyright (2021) Elsevier Ltd.

In addition to the above-mentioned studies, another type of magnetic core has also been explored as MRI contrast agents for brain tumor imaging. Cu is another important nutrient for humans and also acts as a cofactor for various enzymes. For example, it can help in the absorption and utilization of iron [177]. Interestingly, Cu-based nanomaterials with the capability of shortening T1 relaxation time have recently received increasing attention since they could effectively induce T1WI signal enhancement [178, 179]. Cu is metabolized through liver, in which Cu is mobilized into the external circulation or secreted into the bile for elimination [180]. Notably, Cu ions would greatly contribute to intratumoral Fenton-like catalyzation process with generation of large amount of reactive oxygen species for killing tumor cells [181]. Recently, a multifunctional core-shell tecto dendrimers with acetyl termini (M-CSTD.NHAc) was proposed to chelate Cu^2+^ for theranostics of orthotopic glioma [99]. Briefly, β-cyclodextrin (CD)-modified G5 PAMAM dendrimers were selected as cores and adamantane (Ad)-functionalized G3 PAMAM dendrimers (G3. NH_2_-Ad) were selected as shells, followed by pyridine modification and Cu^2+^ complexation, respectively (Fig. 9a). The obtained M-CSTD.NHAc/Cu(II) could perform T1WI on orthotopic mouse glioma with a calculated R_1_ of 0.7331 mM^− 1^s^− 1^ (Fig. 9b). Moreover, Cu^2+^ could also allow an efficient Fenton-like reaction by sequentially reacting with intratumoral GSH and H_2_O_2_ for CDT of glioma, leading to tumor cell cycle arrest and cell apoptosis. Compared with the control group, the survival ratio of M-CSTD. NHAc/Cu(II)-treated mice was dramatically increased (Fig. 9c). Meanwhile, treatment with M-CSTD.NHAc/Cu(II) caused significant inhibition of glioma growth, which was reflected in the HE staining images of brain tissues (Fig. 9d). Due to the high efficiency in imaging and therapy, CSTD-based nanoplatform are expected to increasing interest in different areas in the near future. Besides, Cu_2_(OH)PO_4_@PAA NPs have also been reported for T1WI and PTT of glioma [103]. Taken together, Cu-based NPs could act as theranostic nanoplatforms to exert both MRI and CDT of brain tumors.

## Perspectives and conclusion

This review summarizes the state-of-the-art MRI contrast agents for brain tumors diagnosis, which include iron oxide NPs, and Mn-, Gd-, ^19^F- and Cu-based NPs. In order to improve the NP accumulation at the tumor sites and increase the signal-to-background ratio, these NP based MRI contrast agents are generally decorated with specific targeting ligands to cross the BBB in a non-invasive way and maximally enrich in brain tumors with reduced nonspecific uptake.

Although tremendous published research papers have claimed that the developed MRI contrast agents hold great promise for future clinic applications, it should be noticed that there are still some obstacles to translate from bench to bed-side. The first typical obstacle is that the long-term safety especially for the contrast agents containing heavy metal should be thoroughly examined, although most reported MRI contrast agents exhibited no obvious biological cytotoxicity in a short period in vitro and in vivo. To reduce the long-term cytotoxicity, there are several strategies could be considered as follows: (I) Design of contrast agents with strong capability in BBB crossing, brain tumor targeting, long-term circulation, and high MRI sensitivity, is feasible to achieve high imaging fidelity using a relatively low diagnostic threshold dosage and thus decrease the cytotoxicity in vivo; (II) Usage of elements contained in human bodies, such as iron, Mn and Cu, to yield contrast agents and further application of the contrast agents within a safety dosage, contribute to fulfill the requirements in clinic translation; (III) Formulation of contrast agents which are degradable in vivo could, to a certain degree, alleviate the long-term cytotoxicity concern; (IV) Production of MRI contrast agents, which exhibit ultra-small size (e.g., < 5.5 nm) in vivo and thus could be excreted by renal systems, is promising to significantly improve the safety in both short and long term [182]; and (V) To understand how the different NPs affect cellular anabolic or catabolic processes in the long run and summarize the relationship between the NP composition and the in vivo cytotoxicity performance, would greatly assist to design more applicable MRI contrast agents for clinic brain imaging [183, 184]. The second obstacle to prevent the reported MRI contrast agents going to clinic translation, is that the researchers mainly focused on study of the metabolic and excretion pathways of intermediate magnetic cores, but left alone the final NP product containing the magnetic cores. However, the magnetic cores and other components to construct the nano-sized MRI contrast agents may dissociate in the biological environment and distributed in different locations in vivo. Subsequently, the dissociated elements would exhibit different metabolic and excretion pathways in vivo [185]. Therefore, it is strongly suggested to further study the in vivo metabolic and excretion pathways for the NP contrast agents and their dissociated elements with the corresponding tracing technologies, such as FI, MRI, radiolabeling method, and etc., [186]. The third obstacle to delay the clinic translation for the reported MRI contrast agents is that they should be further examined regarding to their imaging, therapy and toxicity performance on non-human primate modals, instead of only on mice models. As known, the physiological environment in mice is far different from the human beings, leading to a huge gap between the experimental and clinic conditions. Overall, future research should pay more attention to solve these aforementioned problems, while developing new MRI contrast agents, and this would expedite the clinic translation of the MRI contrast agents.

Despite MRI possesses a lot of inherent advantages in clinical diagnosis of brain tumors, single MRI contrast agent is unable to satisfy the growing medical demands. Therefore, the development of novel nanoplatforms that integrate diverse diagnostic and therapeutic abilities are a major trend for the future. Simply put, we hope this review will inspire great interest from researchers in different areas to participate in establishing multifunctional MRI contrast agents-based nanoplatforms as highly efficient brain cancer theranostics.

## Abbreviations

AMT: Adsorptive-mediated transcytosis
BBB: Blood–brain barrier
BTB: Blood-tumor barrier
CDT: Chemodynamic therapy
CMT: Carrier-mediated transcytosis
CT: Computed tomography
Cu: Copper
ECs: Endothelial cells
EPR effect: The enhanced permeability and retention effect
FI: Fluorescence imaging
FUS: Focused ultrasound
Gd: Gadolinium
GBM: Glioblastoma
HA: Hyaluronic acid
HE staining: Hematoxylin and eosin staining
LRP1: Low-density lipoprotein receptor protein 1
MBs: Microbubbles
Mn: Manganese
MRI: Magnetic resonance imaging
MSCs: Mesenchymal stem cells
MT: Magnetic targeting
NIR-I: The first near-infrared
NIR-II: The second near-infrared
NPs: Nanoparticles
NSCs: Neural stem cells
PAI: Photoacoustic imaging
PET: Positron emission tomography
PTT: Photothermal therapy
RMT: Receptor-mediated transcytosis
SPECT: Single photon emission CT
SPIONs: Superparamagnetic iron oxide NPs
T1WI: T1-weighted MRI
T2WI: T2-weighted MRI
TEM: Transmission electron microscope
TJs: Tight junctions
TME: Tumor microenvironment
US: Ultrasound
USPIONs: Ultrasmall superparamagnetic iron oxide NPs
